# Case of Death from Chloroform

**Published:** 1867-01

**Authors:** C. R. Parke

**Affiliations:** Bloomington, Ill.


### ARTICLE VI.

### CASE OF DEATH FROM CHLOROFORM.

By. C. R. PARKE, M.D., Bloomington, Ill.

Read to the Illinois State Medical Society, June, 1866.

MR. PRESIDENT:—Permit me to report the following case of death from the inhalation of chloroform:—

On Saturday, June 2d, I was called to administer chloroform to Miss ——, age about 20 years, apparently in good health. Object, the extraction of teeth.

On the previous Wednesday, chloroform had been administered by a dentist, and six molar teeth extracted, without any deleterious effects.

On Saturday, she seated herself in a regular dental chair, was quite cheerful, and anxious to get under the influence of chloroform. I placed about $\frac{1}{3}$ of a drachm of chloroform on a sponge and applied it to the nose in the usual way, covered by a towel. This quantity not being sufficient to produce the desired result, an additional $\frac{1}{3}$ of a drachm was poured upon the sponge, which, amount, in a few minutes, sufficiently affected her so as to enable the dentist to extract three molar teeth, with but little pain. She sat up and spit out the blood that accumulated in the mouth, after which she gave me to understand that I must give her more chloroform before she could submit to the further extraction of any more teeth. The remainder of the *drachm* of chloroform was then poured upon the sponge and given as before. In a few minutes, she was considered sufficiently under its influence, to have the remaining teeth (three) extracted.

Just before the doctor succeeded in extracting the last tooth, I noticed a deathly pallor of the countenance; complete cessation of respiration; pulse scarcely perceptible for a minute, when it ceased entirely. There was also considerable capillary congestion about the skin of the upper portion of the chest, also dilatation of the pupils. Prior to the appearance of the pallor of the face, there was not an unfavorable symptom present.

Immediately upon the presentation of the above symptoms, I drew the tongue as far out of the mouth as was necessary to allow the air to enter freely, and, with the assistance of Dr. H. Luce, kept up artificial respiration. Applied aqua ammonia to the nostrils and introduced a small quantity of brandy into the mouth. A catheter was also introduced down into the pharynx, through which we blew, thinking the air might the more readily enter the lungs; at the same time we applied the electro-galvanic battery to the spine and muscles of respiration, all of which means were used diligently for three-quarters of an hour without avail, she only making three attempts at inspiration during the commencement of our efforts at artificial respiration. The pulse acted as it commonly does, during the administration of chloroform, first being excited, then depressed, nothing indicating cardiac trouble of any kind. There was none of that lividity of countenance witnessed in asphyxia. The whole transaction, up to the fatal minute, was not over twenty-five minutes; and the amount of chloroform used, only one drachm.

This is the first fatal case I ever witnessed from the inhalation of chloroform, and I suppose I have administered it, and seen it administered, in this country and in Europe, nearly a thousand times.

Any suggestions thrown out by this Society, as to the particular action of the anæsthetic, and the cause of death in this case, will be thankfully received. My opinion is, it was paralysis of the heart, with congestion of lungs.

